# Efficient Second-Harmonic Generation in Adapted-Width Waveguides Based on Periodically Poled Thin-Film Lithium Niobate

**DOI:** 10.3390/mi15091145

**Published:** 2024-09-12

**Authors:** Junjie He, Lian Liu, Mianjie Lin, Houhong Chen, Fei Ma

**Affiliations:** School of Physics, Sun Yat-sen University, Guangzhou 510275, China; hejj77@mail2.sysu.edu.cn (J.H.); liulian9@mail2.sysu.edu.cn (L.L.); linmj33@mail2.sysu.edu.cn (M.L.); chenhh73@mail2.sysu.edu.cn (H.C.)

**Keywords:** thin-film lithium niobate, second harmonic generation, dispersion engineering

## Abstract

Frequency conversion process based on periodically poled thin-film lithium niobate (PPTFLN) has been widely recognized as an important component for quantum information and photonic signal processing. Benefiting from the tight confinement of optical modes, the normalized conversion efficiency (NCE) of nanophotonic waveguides is improved by orders of magnitude compared to their bulk counterparts. However, the power conversion efficiency of these devices is limited by inherent nanoscale inhomogeneity of thin-film lithium niobate (TFLN), leading to undesirable phase errors. In this paper, we theoretically present a novel approach to solve this problem. Based on dispersion engineering, we aim at adjusting the waveguide structure, making local waveguide width adjustment at positions of different thicknesses, thus eliminating the phase errors. The adapted waveguide width design is applied for etched and loaded waveguides based on PPTFLN, achieving the ultrahigh power conversion efficiency of second harmonic generation (SHG) up to 2.1 × 10^4^%W^−1^ and 6936%W^−1^, respectively, which surpasses the power conversion efficiency of other related works. Our approach just needs standard periodic poling with a single period, significantly reducing the complexity of electrode fabrication and the difficulty of poling, and allows for the placing of multiple waveguides, without individual poling designs for each waveguide. With the advantages of simplicity, high production, and meeting current micro–nano fabrication technology, our work may open a new way for achieving highly efficient second-order nonlinear optical processes based on PPTFLN.

## 1. Introduction

As the “silicon” in nonlinear optics, lithium niobate (LN) is widely recognized as one of the most popular optical materials because of its large second-order nonlinear coefficients (d_33_ = 27.4 pm/V) and wide transparency window (350 nm–4.5 μm) [1]. Extensive research in second-order nonlinear optics, including SHG, sum frequency generation (SFG), and difference frequency generation (DFG) have been conducted in LN-based devices [2,3,4]. Efficient nonlinear frequency conversion processes must fulfill phase matching condition. Among different methods to address this issue, quasi-phase matching (QPM) is a favorite technique because of some prominent advantages, such as being applicable to arbitrary second-order nonlinear processes and utilizing the largest second-order nonlinear coefficient d_33_. The ferroelectric domains of LN can be periodically reversed for QPM, thus forming periodically poled LN [5,6,7]. Constructing waveguides on LN is another key to achieving high conversion efficiency. Traditional bulk LN waveguides based on proton exchange [8] or titanium diffusion [9] can only provide a small core-cladding refractive index difference (Δn~0.02), resulting in large modal area and reduction in nonlinear interaction strength. Consequently, in order to achieve high conversion efficiency, these bulk devices necessitate longer interaction length, which brings a significant challenge for the fabrication of compactly integrated photonics platforms. In recent years, with the increasing demands for device miniaturization and integration, TFLN has emerged as an exceptional platform for research and the applications of nonlinear optics [10,11,12]. Due to the nanophotonic waveguide based on TFLN exhibiting a tight confinement in the modal field, it can significantly enhance the interaction between light and matter; thus, the NCE is increased by more than an order of magnitude in comparison to their bulk counterparts [13,14]. Recently, combining with periodic poling technique, various efficient nonlinear frequency conversion processes have been realized in compact nanophotonic waveguides, including loaded waveguides [15,16] and etched waveguides [17,18,19].

Although these PPTFLN waveguides exhibit NCE (unit: %W^−1^cm^−2^) more than 20 times higher than that of traditional bulk LN waveguides, their power conversion efficiency (unit: %W^−1^) remains lower than the reported values from the state-of-the-art bulk devices. This is mainly because of the inherent nanoscale inhomogeneity in TFLN; for a 600 nm thick LN layer, its thickness deviation of ±15 nm is inevitably introduced during TFLN manufacturing process, which limits the coherent interaction length of second-order nonlinear processes to only a few millimeters. Unlike bulk LN waveguides, the effective refractive index of waveguide mode in TFLN is more sensitive to the variation of waveguide structural parameters, which affects the phase-matching condition, consequently leading to random phase errors. Yet as phase errors accumulate along the waveguide, which hinders the further enhancement of power conversion efficiency. Recently, Zhao et al. compared the impact of etched depth, waveguide width, and inhomogeneity of thickness on QPM spectrum, where the inhomogeneity of thickness is the primary cause of the mismatched QPM spectrum [20]. Moreover, it introduces some unfavorable impacts, including broadened central peaks and undesirable side lobes, resulting in reduced conversion efficiency. Therefore, to enable the waveguide on TFLN to truly outperform their bulk counterparts, achieving efficient nonlinear frequency conversion processes and enhancing power conversion efficiency, is imperative.

Aiming at this problem, Chen et al. employed an adapted poling design to overcome the inherent nanoscale inhomogeneity of TFLN and achieve a power conversion efficiency of SHG up to 10^4^%W^−1^, approaching the theoretical performance of LN nanophotonic waveguides. Furthermore, they demonstrate LN nanophotonic waveguides that outperform conventional crystals in all critical merits, including nonlinear efficiency, power consumption, optical bandwidth, and wavelength tunability [21]. However, utilizing adapted poling approach necessitates the design of a precise poling period at different thicknesses positions, which is extremely challenging for electrode fabrication and domain inversion. In other words, it is not easy to achieve the minimal variation of a poling period and maintain the optimal 50% duty cycle for so many complex and irregular poling periods. Moreover, once adapted poling can only accommodate one waveguide, resulting in low production.

In this work, we theoretically propose a novel approach to compensate for the inherent nanoscale inhomogeneity of TFLN. By employing dispersion engineering, the phase errors caused by thickness inhomogeneity of TFLN can be eliminated, thereby leading to a further constructive enhancement in power conversion efficiency. For the etched waveguide based on PPTFLN, we employ an adapted waveguide width design, achieving high power conversion efficiency of SHG up to 2.1 × 10^4^%W^−1^, which is comparable to the performance of adapted poling design. Subsequently, in order to further simplify the fabrication process, we utilized the same design on an unetched loaded waveguide based on PPTFLN, achieving a power conversion efficiency of 6936%W^−1^, which surpassed the power conversion efficiency of reported loaded waveguides based on PPTFLN. As the adapted waveguide width approach utilizes the standard periodic poling with single period, it avoids aperiodic poling design, significantly reducing the complexity of electrode fabrication and the difficulty of poling. In addition, compared to adapted poling design, our approach allows for the placing of multiple waveguides when the poling gap between the opposite electrodes is large, without individual poling designs for each waveguide. With the advantages of simple design, high production, and utilizing current micro–nano fabrication technology, our proposed approach provides a new avenue for achieving highly efficient second-order nonlinear optical processes on TFLN and would significantly improve the corresponding applications.

## 2. Theory and Design

### 2.1. Adapted Waveguide Width Design in Etched Waveguide

In the past, due to the assumption that the thickness of TFLN is uniform when designing waveguide structures, a uniform and single poling period is usually employed for QPM in SHG, ensuring that fundamental frequency (FF) and second harmonic (SH) waves satisfy phase-matching conditions along the entire waveguides. However, the inhomogeneity of thickness can introduce unneglectable impact on QPM. The poling period Λ can be expressed as [22]:

Λ = λ_ω_/2(n_2ω_ − n_ω_) (1)

where n_ω_ and n_2ω_ are effective refractive index of FF and SH waves, respectively, and λ_ω_ is FF wavelength. The effective refractive index of etched waveguide depends on the waveguide structural parameters, such as the thickness of TFLN *t*, etched depth *h*, etched sidewall angle *θ*, and waveguide width *w*. If the other parameters of waveguide structure remain constant, only *t* changes, i.e., the thickness of slab changes, still leading to the change of the effective refractive index of FF and SH waves. According to Equation (1), the required poling period Λ varies accordingly. Therefore, the phase errors appear at the positions of inhomogeneous thickness, with the accumulation of phase errors along the waveguide, which significantly restricts the power conversion efficiency of SHG. In the case of perfect poling, for a lossless waveguide without pump depletion, the power conversion efficiency of SHG, $\eta_{\mathrm{SHG}}$, can be expressed as [15]:

(2) $$\eta_{\mathrm{SHG}}=\eta_{\mathrm{nor}}L^{2}{sinc}^{2}(L\Delta k/2)$$

where $\eta_{\mathrm{nor}}$ is the normalized conversion efficiency, *L* is the length of waveguide, and $\Delta k=\frac{4\pi }{\lambda_{\omega }}(n_{2\omega }-n_{\omega })-\frac{2\pi }{\Lambda }$ represents the phase mismatch after periodic poling. The effective refractive index of FF and SH waves keep constant along a fixed-width waveguide without considering the inhomogeneity of TFLN thickness. When choosing a right single poling period Λ, making Δk=0, the power conversion efficiency is enhanced with the increase of waveguide length. However, since the effective refractive index of FF and SH waves actually changes owing to the inhomogeneity of TFLN thickness, a single poling period cannot make sure that Δk always equals zero along the entire waveguide. In other words, in the condition of Δk≠0, with the accumulation of phase errors along the waveguide, it will significantly restrict the power conversion efficiency of SHG; that is the reason why the waveguide length was generally limited to ~5 mm for the fixed-width waveguide. With the utilization of adapted waveguide width design with single-period poling, or adapted poling design with fixed-width waveguide, the phase errors can be eliminated along the waveguide, always achieving perfect phase-matching. Under this condition (Δk=0), Equation (2) is equivalent to:

(3) $$\eta_{\mathrm{SHG}}=\eta_{\mathrm{nor}}L^{2}=\frac{32}{\varepsilon_{0}cn_{\omega }^{2}n_{2\omega }\lambda_{\omega }^{2}}\frac{\xi^{2}d_{33}^{2}}{A_{\mathrm{eff}}}L^{2}$$

where $\varepsilon_{0}$ and c are, respectively, permittivity and light speed in a vacuum and d_33_ is the nonlinear coefficient of lithium niobate. ξ is the spatial modal field overlap between FF and SH waves, defined as:

(4) $$\xi =\frac{\int_{\chi^{\left(2\right)}}{(E_{\omega }^{*}(x,z))}^{2}E_{2\omega }(x,z)dxdz}{{\left|\int_{\chi^{\left(2\right)}}{\left|E_{\omega }\right|}^{2}E_{\omega }dxdz\right|}^{\frac{2}{3}}{\left|\int_{\chi^{\left(2\right)}}{\left|E_{2\omega }\right|}^{2}E_{2\omega }dxdz\right|}^{\frac{1}{3}}}$$

where $\int_{\chi^{\left(2\right)}}$ denotes two-dimensional integration over the lithium niobate thin film. $E_{\omega }\left(x,z\right)$ and $E_{2\omega }\left(x,z\right)$ are, respectively, the electric fields of the TE bound mode of FF and SH waves. In Equation (2), $A_{\mathrm{eff}}\equiv {\left(A_{1}^{2}A_{2}\right)}^{\frac{1}{3}}$ is the effective modal area with

(5) $$A_{i}=\frac{{\left(\int_{\mathrm{all}}{\left|E_{i}\right|}^{2}dxdz\right)}^{3}}{{\left|\int_{\chi^{\left(2\right)}}{\left|E_{i}\right|}^{2}E_{i}dxdz\right|}^{2}},\;(i=\omega ,2\omega )$$

where $\int_{\mathrm{all}}$ denotes two-dimensional integration over all space.

**Figure 1:**
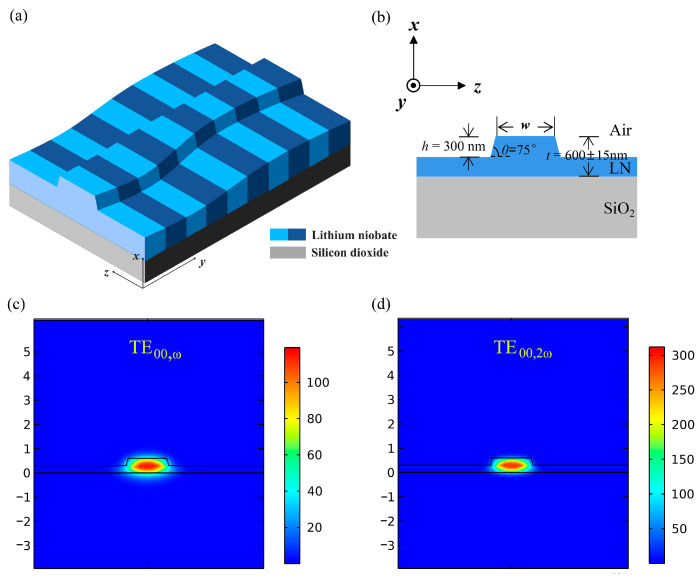
(**a**) Three-dimensional structure diagram of etched waveguide based on PPTFLN with adapted waveguide width design. (**b**) Schematic diagram depicting the *z*-*x* cross section of waveguide structure, where *w* and *t* denote the width and thickness of the etched waveguide, respectively. The etched depth and sidewall angle are *h* = 300 nm and *θ* = 75°, respectively. The modal field distribution of TE bound mode for (**c**) FF wave at 1600 nm and (**d**) SH wave at 800 nm.

Unlike the adapted poling design, we aim at adjusting the waveguide structure, making local structural adjustment at positions of inhomogeneous thickness. Thereby, it eliminates the phase errors and enables the achievement of optimal QPM throughout the entire waveguide. Figure 1a,b are the three-dimensional schematic and cross-sectional illustration of the proposed waveguide, respectively. The length of the proposed waveguide is *L* = 21 mm. The TFLN consists of a X-cut lithium niobate thin film with a thickness of *t* = 600 ± 15 nm and a 2 μm thick silicon dioxide layer. The etched depth and sidewall angle are *h* = 300 nm, and *θ* = 75°, respectively, and *w* is the waveguide width. In X-cut TFLN, TE modes are often chosen to utilize the maximum nonlinear coefficient d_33_, thus enhancing the SHG efficiency. The modal field distribution of TE fundamental modes for FF wave at 1600 nm and SH wave at 800 nm are shown in Figure 1c,d.

**Figure 2:**
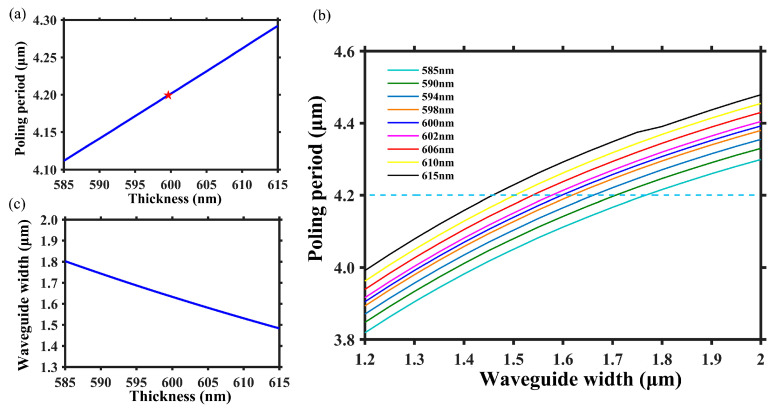
(**a**) The relationship between poling period and LN thickness when the other waveguide structural parameters are fixed. The red star represents the required poling period when LN thickness is 600 nm, and the blue line represents the required poling period as LN thickness changes. (**b**) The variation of poling period with waveguide width under different LN thicknesses. (**c**) The required waveguide width as a function of LN thickness when poling period sets as Λ = 4.20 μm.

Figure 2a illustrates the dependency of poling period on LN thickness while the other waveguide structural parameters are fixed, i.e., *h* = 300 nm, *θ* = 75°, and *w* = 1.60 μm. When the LN layer thickness *t* = 600 nm, the poling period Λ is 4.20 μm, and if the thickness changes by 30 nm, the corresponding variation of poling period is 180 nm. The change in poling period is primarily attributed to the variation in effective refractive index of FF and SH waves, which results from inhomogeneous thickness of LN; thus, adjusting the waveguide width allows for the manipulation of the effective refractive index of FF and SH waves, ensuring a uniform poling period for QPM at positions of inhomogeneous thickness. Figure 2b illustrates the relationship between poling period and waveguide width under different thicknesses of LN. Evidently, adjusting the waveguide width allows for the manipulation of the required poling period, achieving optimal QPM with a constant Λ at different LN thicknesses. Figure 2c shows the required waveguide width for different LN thicknesses at the constant poling period Λ = 4.20 μm. When the LN thickness changes by 30 nm, the corresponding waveguide width changes by 320 nm. Therefore, in order to address the phase errors resulting from inherent nanoscale inhomogeneity of TFLN, it is possible to locally adjust the structure at position with different LN thicknesses and designs corresponding waveguide width to achieve uniform effective thickness of LN. This enables optimal QPM for FF and SH waves along the entire waveguide.

**Figure 3:**
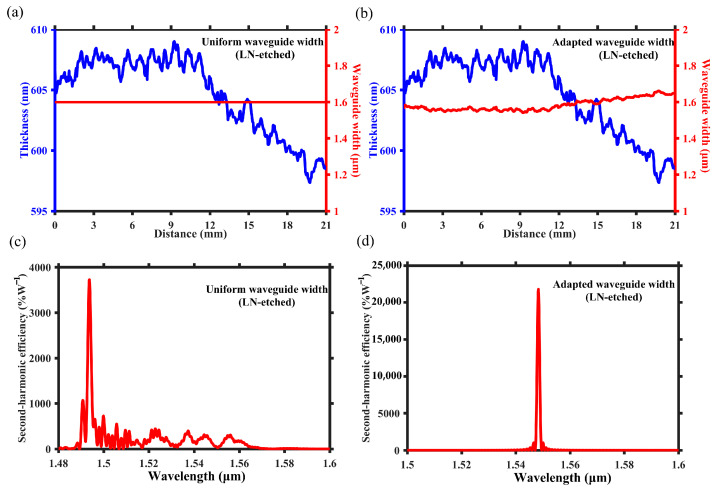
(**a**,**b**) The blue curves show the distribution of LN thickness along the etched waveguide. The red lines show (**a**) uniform waveguide width design and (**b**) adapted waveguide width design along the etched waveguide, respectively. (**c**,**d**) are the SH spectrum corresponding to uniform and adapted waveguide width design, respectively.

As depicted in Figure 3a,b, the blue curves represent LN thickness along the waveguide direction with the distance of 21 mm and the specific data are based on the measured results reported by Chen et al. [21]. The thickness data along the waveguide are accessed with a step size of 50 μm. As the waveguide length is 21 mm, the adapted-width waveguide is actually composed of 420 sections. Each section is 50 μm long, and its width is set with the precision of 10 nm scale in the simulation. As the red lines shown, Figure 3a,b depict two designs featuring uniform and adapted waveguide width, respectively, with the same and single poling period of 4.20 μm. Due to the required poling period varies with LN thickness, the etched waveguide based on PPTFLN with uniform waveguide width demonstrates significant deviation from the theoretical sinc^2^ function in its SH spectrum. As shown in Figure 3c, the center wavelength moves to shorter, the simulated peak efficiency is 3724%W^−1^, and the number of side lobes and peaks increases. However, by employing the adapted waveguide width design based on Figure 2c, a locally structural adjustment is made to the positions of different thicknesses, thus solving the problem of phase errors. Figure 3d illustrates that its SH spectrum aligns with the theoretical sinc^2^ function. In comparison to uniform waveguide width design, it demonstrates higher peak efficiency and narrower bandwidth, with the simulated peak efficiency of 21,780%W^−1^, which indicates that the inherent nanoscale inhomogeneity of TFLN can be addressed through adapted waveguide width design.

For the adapted waveguide width design, waveguide width varies with TFLN thickness. It is necessary to evaluate whether the change in waveguide width would cause additional loss for the entire waveguide, although the change is very small. In the designed etched waveguide, the minimum and maximum waveguide widths are 1.54 μm and 1.66 μm, respectively, where the difference of effective refractive index is the largest. We assume that the waveguide width periodically alters between 1.54 μm and 1.66 μm throughout the entire waveguide with a length of 21 mm, and the simulated transmission is 95% for FF wave. However, the real transmission is obviously larger than 95%, because the waveguide width difference between adjacent sections is below 20 nm, which is much smaller than that of the hypothetical waveguide. Therefore, the adapted waveguide width design in the etched waveguide does not introduce significant additional loss and the transmission of waveguide is still maintained at a high level.

The fabrication of etched waveguide based on PPTFLN necessitates the direct etching of LN, requiring expensive equipment and intricate processes, and directly impacts the propagation loss. Moreover, the effective refractive index of FF and SH waves are very sensitive to the variation of waveguide width with the adjustable range of only 320 nm, which requires higher precision in the fabrication of waveguide width. Therefore, to further reduce the difficulty in fabrication, we apply the adapted waveguide width design to loaded waveguide.

### 2.2. Adapted Waveguide Width Design in Loaded Waveguide

**Figure 4:**
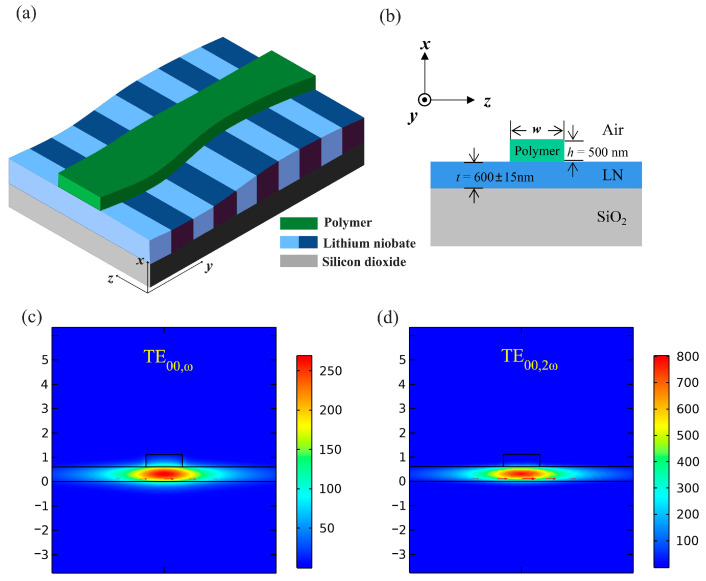
(**a**) Three-dimensional structure diagram of the polymer-loaded waveguide based on PPTFLN with an adapted waveguide width design. (**b**) Schematic diagram depicting the *z*-*x* cross section of waveguide structure, where w denotes the width of the polymer-loaded waveguide. The modal field distribution of TE-bound mode for (**c**) the FF wave at 1600 nm and (**d**) the SH wave at 800 nm.

By adding polymers to TFLN, it can form a region in which the effective refractive index is higher than that in the regions without polymers, thereby forming an optical waveguide. Figure 4a,b are the three-dimensional schematic and cross-sectional illustration of the proposed polymer-loaded waveguide with adapted waveguide width design, respectively. The length of the proposed waveguide is *L* = 21 mm. The effective refractive index of loaded waveguide depends on the thickness of LN layer *t*, the thickness of polymer layer *h*, and the polymer waveguide width *w*. The polymer (ARP6200) layer with a thickness of *h* = 500 nm is spun on TFLN, then exposed and developed to form a waveguide with a width of *w*. The TFLN consists of an X-cut lithium niobate thin-film with a thickness of *t* = 600 nm ± 15 nm and a 2 μm thick silicon dioxide layer. Similarly, TE modes are chosen to utilize the maximum nonlinear coefficient d_33_, thus enhancing the SHG efficiency. The modal field distribution of TE fundamental modes for FF wave at 1600 nm and SH wave at 800 nm are shown in Figure 4c,d.

**Figure 5:**
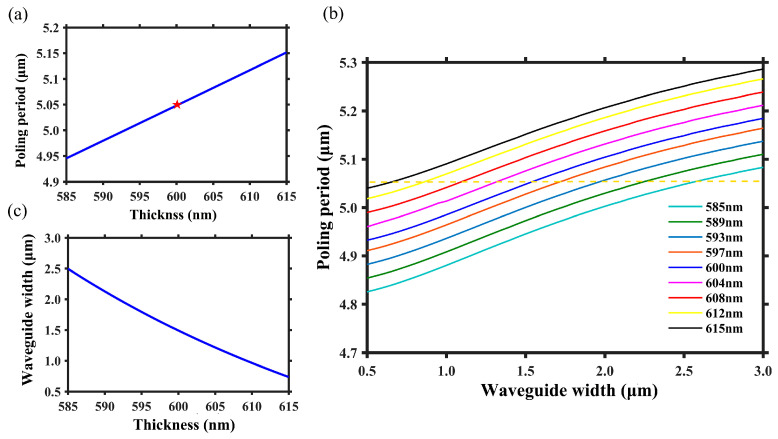
(**a**) The relationship between poling period Λ and LN thickness *t* when the other waveguide structural parameters are fixed. The red star represents the required poling period when LN thickness is 600 nm, and the blue line represents the required poling period as LN thickness changes. (**b**) The variation in poling period with waveguide width under different LN thicknesses. (**c**) The required waveguide width as a function of LN thickness when poling period sets as Λ = 5.05 μm.

Figure 5a shows the dependency of poling period on LN thickness while the other waveguide structural parameters are fixed, i.e., *h* = 500 nm and *w* = 1.50 μm. When the LN layer thickness *t* = 600 nm, the poling period Λ is 5.05 μm, and if the thickness changes by 30 nm, the corresponding variation of poling period is 260 nm. Figure 5b denotes the relationship between poling period and waveguide width under different thicknesses of LN. Obviously, adjusting the waveguide width allows for manipulation of the required poling period, achieving optimal QPM with a constant Λ at different thicknesses of LN. Due to the refractive index of polymer being lower than that of LN, the effective refractive indices of FF and SH waves are relatively insensitive to the variation of waveguide width. Consequently, for the same range of LN thickness variation, the adjustable range of waveguide width is larger in the polymer-loaded waveguide. Figure 5c shows the required waveguide width for different LN thicknesses at the constant poling period Λ = 5.05 μm. When the LN thickness *t* changes by 30 nm, the adjustable range of waveguide width *w* is 1760 nm, which is almost six times larger than that of etched waveguide, thus significantly reducing the fabrication difficulty.

**Figure 6:**
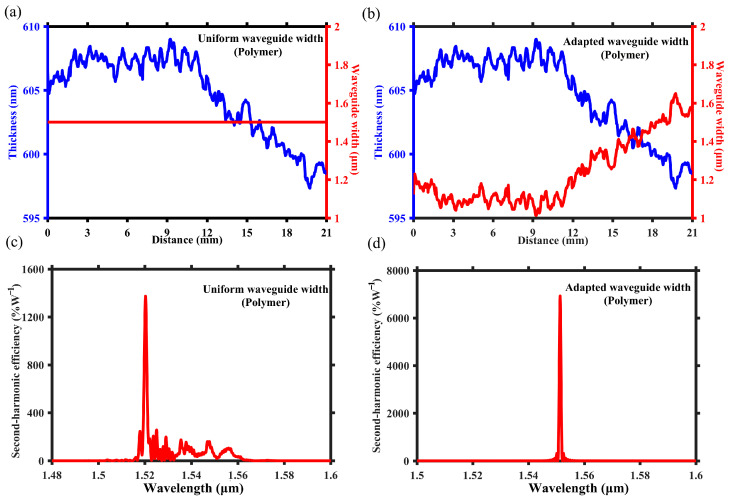
(**a**,**b**) The blue curves show the distribution of LN thickness along the loaded waveguide. The red lines show (**a**) uniform waveguide width design and (**b**) adapted waveguide width design along the loaded waveguide, respectively. (**c**,**d**) are the SH spectrum corresponding to uniform and adapted waveguide width design, respectively.

As the red lines shown, Figure 6a,b depict two designs featuring uniform and adapted waveguide widths, respectively, with the same poling and single period of 5.05 μm. Similarly, due to the inhomogeneity of LN thickness, the polymer-loaded waveguide based on PPTFLN with a uniform waveguide width design demonstrates significant deviation from the theoretical sinc^2^ function in its SH spectrum. As shown in Figure 6c, the uniform waveguide design also exhibits a shift in the central wavelength and an increase in the number of side lobes and side peaks, and the simulated peak efficiency is 1373%W^−1^. Yet after applying the adapted waveguide width design based on Figure 5c, the SH spectrum closely aligns with the sinc^2^ function. Due to the elimination of side lobes and side peaks, as depicted in Figure 6d, the peak efficiency is significantly improved to 6925%W^−1.^ Therefore, the adapted waveguide width design can be also employed to overcome the inherent nanoscale inhomogeneity of TFLN for polymer-loaded waveguides based on PPTFLN. Similarly, for the loaded waveguide, the same method is applied to evaluate the loss. The minimum and maximum waveguide widths are 1.01 μm and 1.64 μm, respectively, where the difference of effective refractive index is the largest. The simulated transmission is 82% when the waveguide width is periodically altered between 1.01 μm and 1.64 μm throughout the entire waveguide. The largest difference of waveguide width between adjacent sections is only 50 nm, which is considerably smaller than that of the assumed waveguide; thus, the actual transmission is obviously much larger than 82%. Consequently, the adapted waveguide width design in loaded waveguide does not introduce significant additional loss either. All simulation results were obtained via model analysis using the COMSOL Multiphysics 5.6 software.

## 3. Result and Discussion

**Figure 7:**
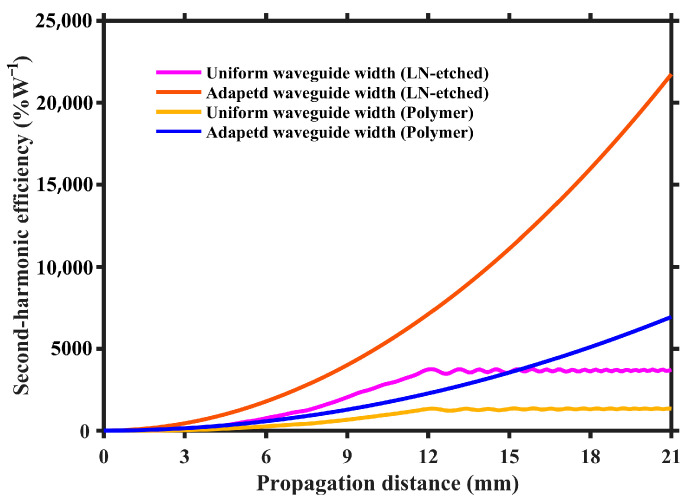
Calculated peak power conversion efficiency of nanophotonic LN waveguides with different lengths using uniform waveguide width and adapted waveguide width design.

Through above simulated results, the adapted waveguide width design in etched and loaded waveguides based on PPTFLN has been shown to effectively overcome the phase errors arising from inherent nanoscale inhomogeneity, thereby enhancing the power conversion efficiency of SHG. However, despite the utilization of adapted waveguide width design in different types of waveguide, there are distinct advantages and disadvantages for etched and loaded waveguides. As illustrated in Figure 7, the power conversion efficiency of the etched waveguide based on adapted waveguide width design significantly surpasses that of the polymer-loaded waveguide by several magnitudes. Yet the etched waveguide necessitates the direct etching of LN, which not only requires expensive equipment but also intricate processes; thus, obtaining a high-quality etched structure is not an easy task. In contrast, a polymer-loaded waveguide does not require the etching of LN, which only needs spin coating, exposure, and development to complete the fabrication of waveguide structure; thereby, the process is straightforward and amenable to mass production.

As shown in Table 1 and Table 2, through the utilization of adapted waveguide width design, the power conversion efficiency of SHG for both etched and loaded waveguides based on PPTFLN are higher than that reported in related works. In contrast to the adapted poling design, the adapted waveguide width design has no need to set complex and unpredictable poling periods, significantly reducing the difficulty of electrode fabrication and domain inversion. Furthermore, the adapted poling design is not conducive to fabricating long ferroelectric domains for accommodating multiple waveguides. As the poling gap between the opposite electrodes increases, the position difference between waveguides is significant, and the variations in LN thickness measured along the waveguide direction are quite different. However, as the poling period is determined by the thickness variation at specific waveguide position, placing the waveguide elsewhere does not fully address the phase errors resulting from the inherent nanoscale inhomogeneity of TFLN. Yet the adapted waveguide width design is not constrained by this problem because the poling period remains uniform, allowing for the waveguide to be placed at different positions by designing structures based on the measured thickness variation, with no impact from the domain length.

## 4. Conclusions

In summary, based on dispersion engineering, we theoretically propose a novel way to solve the issue of the inherent nanoscale inhomogeneity of TFLN. Through employing adapted waveguide width design, the waveguide structure is locally adjusted at the positions of inhomogeneous thickness. Thereby, the undesirable phase errors caused by the inherent nanoscale inhomogeneity of TFLN can be eliminated. Benefiting from this novel approach, the presented etched and loaded waveguides based on PPTFLN in this work achieve the high power conversion efficiencies of SHG of up to 2.1 × 10^4^%W^−1^ and 6936%W^−1^, which are higher than those reported in related works. Due to the utilization of periodic poling with single period, rather than aperiodic poling design, the difficulty of electrode fabrication and poling is greatly reduced. Furthermore, our approach allows for the placing of multiple waveguides when the poling gap between the opposite electrodes is large, without individual poling design for each waveguide. In practice, compared to the standard fabrication process of PPTFLN waveguides, the adapted waveguide width approach can be realized just through adjusting the exposure mask after measuring TFLN thickness variation. The presented approach exhibits the advantages of simplicity and high production, and utilizes current micro–nano fabrication technology, providing a new avenue for realization of long PPTFLN devices with enhanced power conversion efficiency. The significant performance improvement of these second-order nonlinear processes would significantly promote the corresponding applications in quantum information and photonic signal processing, such as up-conversion single-photon detectors, resulting in a detection system with low noise and low power consumption.

## Figures and Tables

**Table 1:** Comparison of the reported etched waveguide based on PPTFLN.

Reference	Method		Length (mm)	Theoretical Normalized Conversion Efficiency (%W^−1^cm^−2^)	Theoretical Power Conversion Efficiency (%W^−1^)
[17]	Fixed-width waveguide	Single-period poling	4	4500	720
[18]	Fixed-width waveguide	Single-period poling	0.6	5500	19.8
[23]	Fixed-width waveguide	Single-period poling	5	4636	1159
[24]	Fixed-width waveguide	Single-period poling	25	476	2975
[21]	Fixed-width waveguide	Adapted poling	21	2388	10,531
This work	Adapted-width waveguide	Single-period poling	21	4939	21,780

**Table 2:** Comparison of the reported loaded waveguide based on PPTFLN.

Reference	Method		Length (mm)	Theoretical Normalized Conversion Efficiency (%W^−1^cm^−2^)	Theoretical Power Conversion Efficiency (%W^−1^)
[15]	Fixed-width waveguide	Single-period poling	4.8	1600	368
[16]	Fixed-width waveguide	Single-period poling	4	1400	224
[25]	Fixed-width waveguide	Single-period poling	4.8	1606	370
This work	Adapted-width waveguide	Single-period poling	21	1570	6925

## Data Availability

The original contributions presented in the study are included in the article, further inquiries can be directed to the corresponding author.
